# 9-Hy­droxy-4,8-dimethyl-12-(piperidin-1-ylmeth­yl)-3,14-dioxatricyclo­[9.3.0.0^2,4^]tetra­dec-7-en-13-one

**DOI:** 10.1107/S1600536811038803

**Published:** 2011-09-30

**Authors:** Mohamed Moumou, Ahmed Benharref, Jean-Claude Daran, Ahmed Elhakmaoui, Moha Berraho

**Affiliations:** a Laboratoire de Chimie Biomoléculaire, Substances Naturelles et Réactivité, URAC 16, Faculté des Sciences Semlalia, BP 2390, Bd My Abdellah,40000 Marrakech, Morocco; b Laboratoire de Chimie de Coordination, 205 route de Narbonne, 31077 Toulouse Cedex 04, France; c Laboratoire de Chimie Bioorganique et Analytique, URAC 22, BP 146, FSTM, Université Hassan II, Mohammedia-Casablanca 20810 Mohammedia, Morocco

## Abstract

The title compound, C_20_H_31_NO_4_, was synthesized from 9α-hy­droxy­parthenolide (9α-hy­droxy-4,8-dimethyl-12-methylen-3,14-dioxa-tricyclo­[9.3.0.0^2,4^]tetra­dec-7-en-13-one), which was isolated from the chloro­form extract of the aerial parts of *Anvillea radiata*. The mol­ecule is built up from fused five-and ten-membered rings with the pipyridin-1-yl-methyl group as a substituent. The ten-membered ring adopts an approximate chair–chair conformation, while the six-membered ring display a chair conformation and the five-membered ring an envelope conformation with the C(H)–C–C(H) atom at the flap. The dihedral angle between the ten-membered ring and the lactone ring is 21.7 (4)°. The mol­ecular conformation is stabilized by an O—H⋯N hydrogen bond and the crystal structure is stabilized by weak inter­molecular C—H⋯O inter­actions.

## Related literature

For background to the medicinal uses of the plant *Anvillea radiata*, see: Abdel Sattar *et al.* (1996 ▶); Bellakhdar (1997 ▶); El Hassany *et al.* (2004 ▶); Qureshi *et al.* (1990 ▶). For the typical conformation of sesquiterpene lactones, see: Watson & Zabel (1982 ▶). For reactivity of this sesquiterpene, see: Hwang *et al.* (2006 ▶). For ring puckering parameters, see: Cremer & Pople (1975 ▶).
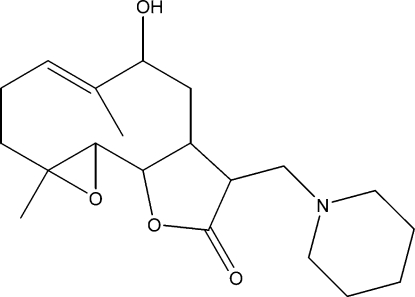


## Experimental

### 

#### Crystal data

C_20_H_31_NO_4_
*M* *_r_* = 349.46
Monoclinic, 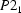

*a* = 11.8390 (6) Å
*b* = 6.7053 (3) Å
*c* = 12.0875 (6) Å
β = 101.399 (5)°
*V* = 940.63 (8) Å^3^
*Z* = 2
Mo *K*α radiation
μ = 0.09 mm^−1^
*T* = 180 K
0.44 × 0.13 × 0.11 mm

#### Data collection

Agilent Xcalibur Sapphire1 long nozzle diffractometer
Absorption correction: multi-scan (*CrysAlis PRO*; Agilent, 2010 ▶) *T*_min_ = 0.858, *T*_max_ = 1.000
10503 measured reflections
2096 independent reflections
1996 reflections with *I* > 2σ(*I*)
*R*_int_ = 0.024

#### Refinement

*R*[*F* ^2^ > 2σ(*F* ^2^)] = 0.028
*wR*(*F* ^2^) = 0.071
*S* = 1.05
2096 reflections
229 parameters
1 restraint
H-atom parameters constrained
Δρ_max_ = 0.16 e Å^−3^
Δρ_min_ = −0.16 e Å^−3^

### 

Data collection: *CrysAlis PRO* (Agilent, 2010 ▶); cell refinement: *CrysAlis PRO*; data reduction: *CrysAlis PRO*; program(s) used to solve structure: *SHELXS97* (Sheldrick, 2008 ▶); program(s) used to refine structure: *SHELXL97* (Sheldrick, 2008 ▶); molecular graphics: *ORTEP-3 for Windows* (Farrugia, 1997 ▶) and *PLATON* (Spek, 2009 ▶); software used to prepare material for publication: *WinGX* publication routines (Farrugia, 1999 ▶).

## Supplementary Material

Crystal structure: contains datablock(s) I, global. DOI: 10.1107/S1600536811038803/bt5649sup1.cif

Structure factors: contains datablock(s) I. DOI: 10.1107/S1600536811038803/bt5649Isup2.hkl

Supplementary material file. DOI: 10.1107/S1600536811038803/bt5649Isup3.cml

Additional supplementary materials:  crystallographic information; 3D view; checkCIF report

## Figures and Tables

**Table 1:** Hydrogen-bond geometry (Å, °)

*D*—H⋯*A*	*D*—H	H⋯*A*	*D*⋯*A*	*D*—H⋯*A*
O4—H4⋯N	0.84	2.12	2.9564 (14)	176
C2—H2⋯O1^i^	1.00	2.45	3.2622 (15)	138
C4—H4*B*⋯O3^ii^	0.99	2.54	3.3387 (16)	137
C6—H6⋯O2^iii^	0.95	2.54	3.2206 (15)	128
C13—H13*A*⋯O2^i^	0.99	2.55	3.5178 (15)	166

Symmetry codes: (i) 
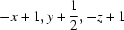
; (ii) 
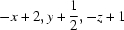
; (iii) 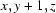.

**Fig. 1.**
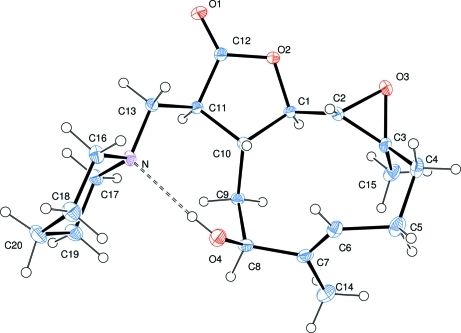
: Molecular structure of the title compound with the atom-labelling scheme. Displacement ellipsoids are drawn at the 30% probability level. H atoms are represented as small spheres of arbitrary radii.

**Fig. 2.**
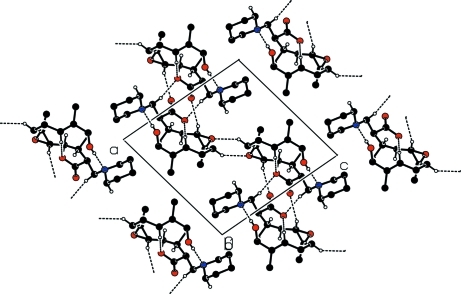
: Packing view showing the C–H···O and O–H···N hydrogen bonds as dashed lines. H atoms not involved in hydrogen bonding have been omitted for clarity.

## supplementary crystallographic information

### Comment

*Anvillea radiata* is a plant that grows in northern Africa and particularly in the two Maghreb countries, Morocco and Algeria. This plant is used in the traditional local medicine for the treatment of dysentery, gastric-intestinal disorders (Bellakhdar, 1997), and hypoglycemic activity (Qureshi *et al.*, 1990), and has been reported to have antitumor activity (Abdel Sattar *et al.*, 1996). In our study of different Moroccan endemic plants, we have demonstrated that the aerial parts of *Anvillea radiata* could be used as a renewable source of 9-hydroxyparthenolide (El Hassany *et al.*, 2004). In order to prepare products with a high added value that can be used in the pharmacology and cosmetics industry, we studied the chemical reactivity of this major constituent of *Anvillea radiata*. Thus, treatment of this sesquiterpene with an equivalent amount of pyridine in ethanol (Hwang *et al.*, 2006) led to 9- hydroxyl-4,8-dimethyl-12-(pipyrydin-1-ylmethyl)- 3,14-dioxatricyclo[9.3.0.02,4]tetradec-7-en-13-one, in a yield of 89%. The structure of this new product was determined by its single-crystal X-ray structure. The molecule contains two fused rings which exhibit different conformations with a pyridin ring as a substituent to the lactone ring. The molecular structure of (I), Fig.1, shows the lactone ring to adopt an envelope conformation, as indicated by Cremer & Pople (1975) puckering parameters Q = 0.2304 (12) Å and φ = 69.5 (3)°. The ten-membered ring displays an approximate chair-chair conformation, while the pyridin ring has a perfect chair conformation with QT = 0.5736 (14) Å, θ = 176.64 (14)° and φ2 = 143 (2)°. This is the typical conformation observed for other sesquiterpenes lactones (Watson & Zabel, 1982). In the crystal structure, the molecules are linked by C—H···O intermolecular hydrogen bonds into zigzag chains along the *a* axis (Fig.2). In addition an intramolecular O—H···N hydrogen bond is also observed.

### Experimental

The mixture of 9α-hydroxyparthenolide (500 mg, 1.98 mmol) and one equivalent of pipyridine in EtOH (20 ml) was stirred for one night at room temperature. The next day the reaction was stopped by adding water (10 ml) and extracted three times with ethyl acetate (3 *x* 20 ml). The combined organic layers were dried over anhydrous MgSO_4_, filtered and concentrated under vacuum to give 584 mg (1.68 mmol) of 9- hydroxyl-4,8-dimethyl-12-(pipyrydin-1-ylmethyl)-3,14- dioxatricyclo[9.3.0.0^2^,^4^]tetradec-7-en-13-one, which was recrystallized in ethyl acetate.

### Refinement

All H atoms were fixed geometrically and treated as riding with C—H = 0.96 Å (methyl), 0.97 Å (methylene), 0. 98Å (methine) with *U*_iso_(H) = 1.2*U*_eq_ (methylene, methine) or *U*_iso_(H) = 1.5*U*_eq_ (methyl, OH). In the absence of significant anomalous scattering, the absolute configuration could not be reliably determined and thus 1747 Friedel pairs were merged and any references to the Flack parameter were removed.

### Crystal data

**Table:**

C_20_H_31_NO_4_	*F*(000) = 380
*M**_r_* = 349.46	*D*_x_ = 1.234 Mg m^−^^3^
Monoclinic, *P*2_1_	Mo *K*α radiation, λ = 0.71073 Å
Hall symbol: P 2yb	Cell parameters from 10503 reflections
*a* = 11.8390 (6) Å	θ = 3.4–26.4°
*b* = 6.7053 (3) Å	µ = 0.09 mm^−^^1^
*c* = 12.0875 (6) Å	*T* = 180 K
β = 101.399 (5)°	Box, colorless
*V* = 940.63 (8) Å^3^	0.44 × 0.13 × 0.11 mm
*Z* = 2	

### Data collection

**Table:**

Agilent Xcalibur Sapphire1 long nozzle diffractometer	2096 independent reflections
Radiation source: fine-focus sealed tube	1996 reflections with *I* > 2σ(*I*)
graphite	*R*_int_ = 0.024
Detector resolution: 8.2632 pixels mm^-1^	θ_max_ = 26.4°, θ_min_ = 3.4°
ω scan	*h* = −14→14
Absorption correction: multi-scan (*CrysAlis PRO*; Agilent, 2010)	*k* = −8→8
*T*_min_ = 0.858, *T*_max_ = 1.000	*l* = −15→15
10503 measured reflections	

### Refinement

**Table:**

Refinement on *F*^2^	Primary atom site location: structure-invariant direct methods
Least-squares matrix: full	Secondary atom site location: difference Fourier map
*R*[*F*^2^ > 2σ(*F*^2^)] = 0.028	Hydrogen site location: inferred from neighbouring sites
*wR*(*F*^2^) = 0.071	H-atom parameters constrained
*S* = 1.05	*w* = 1/[σ^2^(*F*_o_^2^) + (0.0473*P*)^2^ + 0.0809*P*] where *P* = (*F*_o_^2^ + 2*F*_c_^2^)/3
2096 reflections	(Δ/σ)_max_ < 0.001
229 parameters	Δρ_max_ = 0.16 e Å^−^^3^
1 restraint	Δρ_min_ = −0.16 e Å^−^^3^

### Special details

**Table:**

Geometry. All s.u.'s (except the s.u. in the dihedral angle between two l.s. planes) are estimated using the full covariance matrix. The cell s.u.'s are taken into account individually in the estimation of s.u.'s in distances, angles and torsion angles; correlations between s.u.'s in cell parameters are only used when they are defined by crystal symmetry. An approximate (isotropic) treatment of cell s.u.'s is used for estimating s.u.'s involving l.s. planes.
Refinement. Refinement of *F*^2^ against ALL reflections. The weighted *R*-factor *wR* and goodness of fit *S* are based on *F*^2^, conventional *R*-factors *R* are based on *F*, with *F* set to zero for negative *F*^2^. The threshold expression of *F*^2^ > σ(*F*^2^) is used only for calculating *R*-factors(gt) *etc*. and is not relevant to the choice of reflections for refinement. *R*-factors based on *F*^2^ are statistically about twice as large as those based on *F*, and *R*- factors based on ALL data will be even larger.

### Fractional atomic coordinates and isotropic or equivalent isotropic displacement parameters (Å^2^)

**Table:**

	*x*	*y*	*z*	*U*_iso_*/*U*_eq_	
C1	0.71467 (12)	0.2598 (2)	0.66934 (12)	0.0185 (3)	
H1	0.7678	0.1867	0.7306	0.022*	
C2	0.78094 (12)	0.3815 (2)	0.60123 (12)	0.0204 (3)	
H2	0.7329	0.4808	0.5510	0.024*	
C3	0.90205 (13)	0.4403 (3)	0.63599 (14)	0.0242 (3)	
C4	0.93406 (14)	0.6394 (3)	0.59414 (15)	0.0320 (4)	
H4A	0.8832	0.6688	0.5206	0.038*	
H4B	1.0144	0.6341	0.5823	0.038*	
C5	0.92313 (15)	0.8072 (3)	0.67800 (17)	0.0341 (4)	
H5A	0.9905	0.8040	0.7413	0.041*	
H5B	0.9226	0.9379	0.6397	0.041*	
C6	0.81409 (13)	0.7844 (2)	0.72347 (14)	0.0265 (3)	
H6	0.7440	0.7997	0.6703	0.032*	
C7	0.80412 (13)	0.7456 (3)	0.82848 (14)	0.0264 (3)	
C8	0.68939 (14)	0.6851 (2)	0.85621 (13)	0.0237 (3)	
H8	0.6920	0.7171	0.9375	0.028*	
C9	0.67106 (13)	0.4587 (2)	0.84106 (12)	0.0202 (3)	
H9A	0.7448	0.3906	0.8720	0.024*	
H9B	0.6143	0.4157	0.8864	0.024*	
C10	0.62893 (11)	0.3886 (2)	0.71874 (11)	0.0172 (3)	
H10	0.6103	0.5093	0.6700	0.021*	
C11	0.52119 (12)	0.2569 (2)	0.70277 (11)	0.0186 (3)	
H11	0.5205	0.1808	0.7738	0.022*	
C12	0.53608 (12)	0.1144 (2)	0.61067 (12)	0.0207 (3)	
C13	0.40794 (12)	0.3691 (3)	0.66791 (12)	0.0216 (3)	
H13A	0.4040	0.4256	0.5916	0.026*	
H13B	0.3434	0.2737	0.6636	0.026*	
C14	0.90140 (16)	0.7399 (4)	0.93004 (17)	0.0431 (5)	
H14A	0.9745	0.7663	0.9063	0.065*	
H14B	0.9044	0.6079	0.9654	0.065*	
H14C	0.8886	0.8417	0.9844	0.065*	
C15	0.97876 (14)	0.3631 (3)	0.74176 (16)	0.0336 (4)	
H15A	1.0573	0.3466	0.7286	0.050*	
H15B	0.9497	0.2342	0.7621	0.050*	
H15C	0.9793	0.4583	0.8034	0.050*	
C16	0.36161 (14)	0.4537 (3)	0.84869 (13)	0.0241 (3)	
H16A	0.4219	0.3604	0.8864	0.029*	
H16B	0.2882	0.3791	0.8291	0.029*	
C17	0.30269 (13)	0.6655 (3)	0.68699 (13)	0.0258 (3)	
H17A	0.2298	0.5903	0.6649	0.031*	
H17B	0.3248	0.7164	0.6174	0.031*	
C18	0.28408 (15)	0.8390 (3)	0.76082 (15)	0.0316 (4)	
H18A	0.2196	0.9219	0.7207	0.038*	
H18B	0.3543	0.9228	0.7760	0.038*	
C19	0.34845 (15)	0.6219 (3)	0.92854 (13)	0.0315 (4)	
H19A	0.4229	0.6924	0.9513	0.038*	
H19B	0.3266	0.5668	0.9974	0.038*	
C20	0.25678 (15)	0.7677 (3)	0.87217 (14)	0.0341 (4)	
H20A	0.2539	0.8833	0.9225	0.041*	
H20B	0.1804	0.7019	0.8583	0.041*	
N	0.39312 (10)	0.5309 (2)	0.74545 (10)	0.0202 (3)	
O1	0.46527 (10)	0.00689 (19)	0.55635 (10)	0.0299 (3)	
O2	0.64460 (9)	0.12085 (16)	0.59253 (9)	0.0211 (2)	
O3	0.86868 (9)	0.2885 (2)	0.55096 (10)	0.0295 (3)	
O4	0.59735 (10)	0.79420 (18)	0.79140 (10)	0.0297 (3)	
H4	0.5376	0.7235	0.7795	0.045*	

### Atomic displacement parameters (Å^2^)

**Table:**

	*U*^11^	*U*^22^	*U*^33^	*U*^12^	*U*^13^	*U*^23^
C1	0.0182 (6)	0.0179 (7)	0.0187 (6)	−0.0003 (6)	0.0017 (5)	−0.0029 (6)
C2	0.0194 (7)	0.0215 (7)	0.0212 (7)	0.0018 (6)	0.0065 (6)	−0.0019 (6)
C3	0.0180 (7)	0.0247 (8)	0.0314 (8)	−0.0006 (6)	0.0081 (6)	−0.0036 (7)
C4	0.0246 (8)	0.0331 (10)	0.0415 (9)	−0.0051 (7)	0.0143 (7)	0.0047 (8)
C5	0.0301 (8)	0.0225 (8)	0.0514 (11)	−0.0055 (7)	0.0119 (8)	0.0035 (8)
C6	0.0237 (7)	0.0155 (7)	0.0401 (9)	0.0000 (6)	0.0060 (6)	0.0004 (7)
C7	0.0242 (7)	0.0188 (7)	0.0344 (8)	−0.0023 (6)	0.0015 (6)	−0.0065 (6)
C8	0.0251 (7)	0.0208 (7)	0.0240 (7)	0.0002 (6)	0.0022 (6)	−0.0054 (6)
C9	0.0217 (7)	0.0215 (7)	0.0169 (7)	−0.0015 (6)	0.0024 (5)	−0.0015 (6)
C10	0.0171 (6)	0.0175 (7)	0.0169 (7)	−0.0008 (5)	0.0033 (5)	−0.0007 (5)
C11	0.0192 (6)	0.0200 (7)	0.0167 (6)	−0.0033 (6)	0.0041 (5)	0.0000 (6)
C12	0.0211 (7)	0.0215 (7)	0.0198 (6)	−0.0024 (6)	0.0047 (5)	−0.0004 (6)
C13	0.0188 (7)	0.0284 (8)	0.0178 (7)	−0.0012 (6)	0.0041 (5)	−0.0021 (6)
C14	0.0302 (9)	0.0549 (13)	0.0404 (10)	−0.0072 (9)	−0.0021 (7)	−0.0102 (10)
C15	0.0217 (7)	0.0288 (9)	0.0470 (10)	0.0011 (7)	−0.0012 (7)	−0.0003 (8)
C16	0.0269 (7)	0.0272 (8)	0.0199 (7)	0.0002 (7)	0.0089 (6)	0.0032 (6)
C17	0.0235 (7)	0.0325 (9)	0.0221 (7)	0.0055 (7)	0.0057 (6)	0.0052 (7)
C18	0.0319 (8)	0.0283 (9)	0.0342 (9)	0.0086 (7)	0.0058 (7)	0.0037 (7)
C19	0.0377 (9)	0.0373 (10)	0.0202 (7)	0.0049 (8)	0.0078 (6)	−0.0008 (7)
C20	0.0344 (9)	0.0408 (10)	0.0290 (8)	0.0092 (9)	0.0108 (7)	−0.0049 (8)
N	0.0188 (6)	0.0247 (6)	0.0178 (6)	0.0011 (5)	0.0057 (4)	0.0031 (5)
O1	0.0277 (6)	0.0321 (7)	0.0293 (6)	−0.0091 (5)	0.0041 (5)	−0.0100 (5)
O2	0.0207 (5)	0.0207 (5)	0.0226 (5)	−0.0025 (4)	0.0058 (4)	−0.0058 (4)
O3	0.0236 (5)	0.0338 (6)	0.0346 (6)	−0.0012 (5)	0.0145 (5)	−0.0100 (6)
O4	0.0244 (5)	0.0211 (6)	0.0431 (6)	0.0033 (5)	0.0051 (5)	−0.0017 (6)

### Geometric parameters (Å, °)

**Table:**

C1—O2	1.4541 (17)	C11—H11	1.0000
C1—C2	1.488 (2)	C12—O1	1.1982 (19)
C1—C10	1.5401 (19)	C12—O2	1.3461 (17)
C1—H1	1.0000	C13—N	1.466 (2)
C2—O3	1.4444 (17)	C13—H13A	0.9900
C2—C3	1.466 (2)	C13—H13B	0.9900
C2—H2	1.0000	C14—H14A	0.9800
C3—O3	1.445 (2)	C14—H14B	0.9800
C3—C4	1.503 (2)	C14—H14C	0.9800
C3—C15	1.506 (2)	C15—H15A	0.9800
C4—C5	1.537 (3)	C15—H15B	0.9800
C4—H4A	0.9900	C15—H15C	0.9800
C4—H4B	0.9900	C16—N	1.4658 (19)
C5—C6	1.508 (2)	C16—C19	1.512 (2)
C5—H5A	0.9900	C16—H16A	0.9900
C5—H5B	0.9900	C16—H16B	0.9900
C6—C7	1.324 (2)	C17—N	1.4699 (19)
C6—H6	0.9500	C17—C18	1.509 (2)
C7—C14	1.509 (2)	C17—H17A	0.9900
C7—C8	1.517 (2)	C17—H17B	0.9900
C8—O4	1.4136 (19)	C18—C20	1.522 (2)
C8—C9	1.540 (2)	C18—H18A	0.9900
C8—H8	1.0000	C18—H18B	0.9900
C9—C10	1.5375 (19)	C19—C20	1.518 (3)
C9—H9A	0.9900	C19—H19A	0.9900
C9—H9B	0.9900	C19—H19B	0.9900
C10—C11	1.5320 (19)	C20—H20A	0.9900
C10—H10	1.0000	C20—H20B	0.9900
C11—C12	1.504 (2)	O4—H4	0.8400
C11—C13	1.523 (2)		
			
O2—C1—C2	107.15 (11)	C10—C11—H11	109.5
O2—C1—C10	105.68 (10)	O1—C12—O2	121.14 (14)
C2—C1—C10	111.55 (12)	O1—C12—C11	128.04 (14)
O2—C1—H1	110.8	O2—C12—C11	110.82 (12)
C2—C1—H1	110.8	N—C13—C11	113.50 (11)
C10—C1—H1	110.8	N—C13—H13A	108.9
O3—C2—C3	59.52 (9)	C11—C13—H13A	108.9
O3—C2—C1	119.86 (13)	N—C13—H13B	108.9
C3—C2—C1	125.56 (13)	C11—C13—H13B	108.9
O3—C2—H2	113.7	H13A—C13—H13B	107.7
C3—C2—H2	113.7	C7—C14—H14A	109.5
C1—C2—H2	113.7	C7—C14—H14B	109.5
O3—C3—C2	59.49 (9)	H14A—C14—H14B	109.5
O3—C3—C4	115.98 (14)	C7—C14—H14C	109.5
C2—C3—C4	116.10 (14)	H14A—C14—H14C	109.5
O3—C3—C15	113.34 (14)	H14B—C14—H14C	109.5
C2—C3—C15	122.85 (14)	C3—C15—H15A	109.5
C4—C3—C15	116.18 (14)	C3—C15—H15B	109.5
C3—C4—C5	111.64 (14)	H15A—C15—H15B	109.5
C3—C4—H4A	109.3	C3—C15—H15C	109.5
C5—C4—H4A	109.3	H15A—C15—H15C	109.5
C3—C4—H4B	109.3	H15B—C15—H15C	109.5
C5—C4—H4B	109.3	N—C16—C19	110.83 (14)
H4A—C4—H4B	108.0	N—C16—H16A	109.5
C6—C5—C4	110.84 (14)	C19—C16—H16A	109.5
C6—C5—H5A	109.5	N—C16—H16B	109.5
C4—C5—H5A	109.5	C19—C16—H16B	109.5
C6—C5—H5B	109.5	H16A—C16—H16B	108.1
C4—C5—H5B	109.5	N—C17—C18	111.51 (12)
H5A—C5—H5B	108.1	N—C17—H17A	109.3
C7—C6—C5	127.93 (16)	C18—C17—H17A	109.3
C7—C6—H6	116.0	N—C17—H17B	109.3
C5—C6—H6	116.0	C18—C17—H17B	109.3
C6—C7—C14	125.94 (16)	H17A—C17—H17B	108.0
C6—C7—C8	121.18 (14)	C17—C18—C20	111.23 (15)
C14—C7—C8	112.72 (15)	C17—C18—H18A	109.4
O4—C8—C7	111.41 (13)	C20—C18—H18A	109.4
O4—C8—C9	111.66 (12)	C17—C18—H18B	109.4
C7—C8—C9	110.39 (13)	C20—C18—H18B	109.4
O4—C8—H8	107.7	H18A—C18—H18B	108.0
C7—C8—H8	107.7	C16—C19—C20	110.39 (13)
C9—C8—H8	107.7	C16—C19—H19A	109.6
C10—C9—C8	115.28 (13)	C20—C19—H19A	109.6
C10—C9—H9A	108.5	C16—C19—H19B	109.6
C8—C9—H9A	108.5	C20—C19—H19B	109.6
C10—C9—H9B	108.5	H19A—C19—H19B	108.1
C8—C9—H9B	108.5	C19—C20—C18	109.95 (14)
H9A—C9—H9B	107.5	C19—C20—H20A	109.7
C11—C10—C9	113.65 (11)	C18—C20—H20A	109.7
C11—C10—C1	102.95 (12)	C19—C20—H20B	109.7
C9—C10—C1	115.52 (12)	C18—C20—H20B	109.7
C11—C10—H10	108.1	H20A—C20—H20B	108.2
C9—C10—H10	108.1	C16—N—C13	111.44 (13)
C1—C10—H10	108.1	C16—N—C17	110.02 (12)
C12—C11—C13	109.57 (11)	C13—N—C17	108.34 (11)
C12—C11—C10	104.07 (11)	C12—O2—C1	111.05 (11)
C13—C11—C10	114.55 (13)	C2—O3—C3	60.99 (9)
C12—C11—H11	109.5	C8—O4—H4	109.5
C13—C11—H11	109.5		

### Hydrogen-bond geometry (Å, °)

**Table:**

*D*—H···*A*	*D*—H	H···*A*	*D*···*A*	*D*—H···*A*
O4—H4···N	0.84	2.12	2.9564 (14)	176
C2—H2···O1^i^	1.00	2.45	3.2622 (15)	138
C4—H4B···O3^ii^	0.99	2.54	3.3387 (16)	137
C6—H6···O2^iii^	0.95	2.54	3.2206 (15)	128
C13—H13A···O2^i^	0.99	2.55	3.5178 (15)	166

Symmetry codes:  (i) −*x*+1, *y*+1/2, −*z*+1; (ii) −*x*+2, *y*+1/2, −*z*+1; (iii) *x*, *y*+1, *z*.
